# Macro-Micro Mechanics of Granular Soils Under Shear Considering Coupled Effects of Particle Size Distribution and Particle Morphology

**DOI:** 10.3390/ma18020428

**Published:** 2025-01-17

**Authors:** Wei Xiong, Jianfeng Wang

**Affiliations:** 1 Department of Architecture and Civil Engineering, City University of Hong Kong, Hong Kong; xiong.wei@my.cityu.edu.hk; 2 Shenzhen Research Institute of City University of Hong Kong, Shenzhen 518000, China

**Keywords:** particle size distribution, particle morphology, spherical harmonic analysis, discrete element method, X-ray micro-computed tomography, granular soils

## Abstract

This paper investigates the effects of particle morphology (PM) and particle size distribution (PSD) on the micro-macro mechanical behaviours of granular soils through a novel X-ray micro-computed tomography (μCT)-based discrete element method (DEM) technique. This technique contains the grain-scale property extraction by the X-ray μCT, DEM parameter calibration by the one-to-one mapping technique, and the massive derivative DEM simulations. In total, 25 DEM samples were generated with a consideration of six PSDs and four PMs. The effects of PSD and PM on the micro-macro mechanical behaviours were carefully investigated, and the coupled effects were highlighted. It is found that (a) PM plays a significant role in the micro-macro mechanical responses of granular soils under triaxial shear; (b) the PSD uniformity can enhance the particle morphology effect in dictating the peak deviatoric stress, maximum volumetric strain, contact-based coordination number, fabric evolution, and shear band formation, while showing limited influences in the maximum dilation angle and particle-based coordination number; (c) with the same PSD uniformity and PM degree, the mean particle volume shows minimal effects on the macro-micro mechanical behaviours of granular soils as well as the particle morphology effects.

## 1. Introduction

Granular soils are widely utilised in geotechnical engineering and exhibit different micro-macro mechanical responses under various loading conditions. Due to the typical characteristics such as heterogeneity, discontinuity, and anisotropy, the mechanical responses of granular materials are complicated [1,2,3]. Under external loadings, particles within the given sample can rotate, translate, and even break, leading to the reconstruction of grain-scale fabric structure [4,5]. Thus, the granular soils’ macro-scale mechanical responses under triaxial shear should consider their grain-scale properties.

Particle morphology (PM), the intrinsic characteristic of granular soils, is multiscale and can be described by the general form (GF) at a large scale, the local roundness (LR) at an intermediate scale, and the surface texture at a small scale. Many studies have revealed the effects of PM on different aspects of macro-micro mechanics of granular soils, such as bearing capacity [6], packing density [7], stiffness [8,9], soil fabric [10,11], stress-dilatancy response [12,13,14,15], crushability [16], and shear band evolution [2,17,18]. Many optical techniques have been utilised and developed to extract the high-resolution PM [7,19,20,21,22,23,24]. Many particle reconstruction techniques have been further developed to reproduce realistic particle morphology based on the extracted particle morphology of granular materials to reconstruct individual particles precisely. Among them, spherical harmonic analysis (SHA) is widely adopted to reconstruct the three-dimensional PM [25,26,27,28,29,30,31,32,33,34,35,36,37,38,39]. By treating spherical harmonic coefficients as the “morphological gene” of granular soils, Xiong and Wang [40] adopted the spherical-harmonic-based principal component analysis (SH-PCA) to generate morphological gene-mutated virtual particles with morphological variance at different length scales.

Particle size distribution (PSD) is a unique and essential characteristic of granular soils. Two parameters, i.e., the uniformity coefficient Cu and the mean particle size D50, are usually utilised to quantify different PSDs. In recent years, many researchers have investigated the effect of particle size and PSD on the mechanical behaviour of granular soils, including stress-strain-strength behaviours and critical state responses [41,42,43,44,45,46,47,48,49,50,51]. However, the coupled effects of PSD and PM on the micro-macro mechanical behaviours of granular soils, especially the contribution of PSD to the particle morphology effect, have not been thoroughly investigated. Although some researchers considered different PM in discussing PSD effects [49], they utilised the simplified non-spherical particles with overall regularity as the morphology descriptors. Thus, this study considered the sophisticated real particles and their derivative morphologically gene-mutated particles.

Due to the experiments’ expensive and time-consuming features, especially for the in situ X-ray micro-tomography (μCT) test, the discrete element method (DEM) is utilised as an alternative tool to bridge the connections between grain-scale properties and macro-scale mechanical behaviours. Many researchers conducted the virtual testing of granular material and further investigated the constitutive behaviour of granular materials with different test conditions, such as drained and undrained biaxial or triaxial tests [1,52,53,54,55,56]. Early DEM adopted spheres with rheology-type rolling resistance [57,58] or simplified non-spherical particles [59,60,61,62,63,64,65] to represent the particle morphology effect of granular soils, which usually failed to describe the evolution of particle kinematics and inter-particle contacts [66]. In this context, modern DEM considered the sophisticated and realistic PM to generate granular soils, leading to a good agreement between experimental and numerical results at both grain- and macro-scale [67,68].

In this paper, a particle reconstruction framework, including the PM extraction by X-ray μCT, morphological gene mutation by SH-PCA, and triaxial tests using DEM, was adopted to discuss the coupled effect of PSD and PM on the micro-macro mechanical behaviours of granular soils. The remainder of this paper is organised as follows. Section 2 introduces the main procedure of the μCT-based DEM technique. Twenty-five virtual samples were generated with a consideration of six PSDs and three morphological gene mutation degrees at the local roundness-level. Section 3 shows the simulation results of the macro-micro mechanical behaviours of granular soils and discusses the coupled effects of PSD and particle morphology on the micro-macro mechanical behaviours of granular soils. Section 4 gives the concluding remarks.

## 2. Methodology

### 2.1. Particle Reconstruction by X-Ray μCT

**Figure 1:**
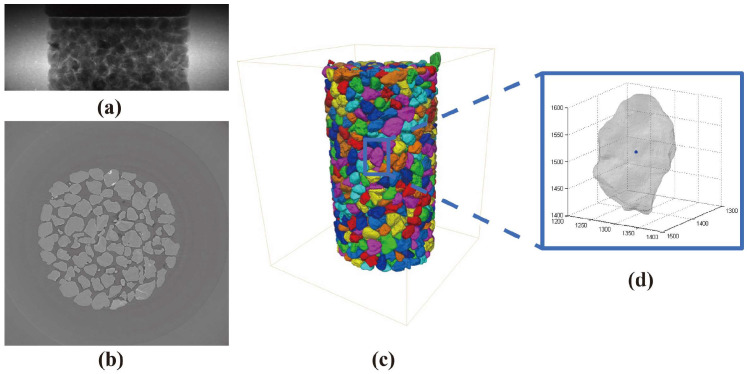
Morphology reconstruction by μCT: (**a**) raw CT projection; (**b**) filtered CT image; (**c**) 3D labelled image; and (**d**) individual particle.

PM was extracted according to the in situ μCT triaxial testing at the BL13HB beamline of the Shanghai Synchrotron Radiation Facility (SSRF). The energy of the X-ray parallel beams was set to 25 Kev, and the resolution of the detector was set to 6.5 μm [22]. A miniature triaxial apparatus was utilised, and the allowed sample size was 8 mm in diameter and 16 mm in height. The testing material was Leighton Buzzard Sand (LBS), which had a particle diameter in a range of 0.6–1.18 mm, and the formed sample contained roughly 1500 grains. Under the external loadings, the upper plate was moving downwards with a constant velocity rate of 0.2%/min, and the confining stress was set to 1.5 Mpa. The axial compression loading was paused for CT scanning at axial strains of 0%, 5%, 10%, and 15%. Note that the PM of all the particles were extracted at the axial strain of 0%. A series of imaging processing techniques were adopted on raw CT projections (Figure 1a) to filter noise (Figure 1b) [69,70], segment particles in contact (Figure 1c) [71], and further extract individual particle boundaries (Figure 1d) (by MATLAB function *bwprim*). Detailed descriptions of the experimental setup and image processing procedures can refer to Cheng and Wang [22]. Here, each μCT-reconstructed particle within the given sample was expressed by 3D point clouds.

### 2.2. Morphological Variance by SH-PCA

The spherical harmonic analysis (SHA) [72], as shown in Equations (1)–(3), was implemented to extract SH coefficients $C_{n}^{m}$ for each particle. With $C_{n}^{m}$ of all particles, SH-PCA was adopted to generate derivative particle morphologies with different degrees of morphological gene mutation as shown in Equations (4) and (5).

(1) $$r(\theta ,\varphi )=\sum_{n=0}^{\infty }\sum_{m=-n}^{n}C_{n}^{m}Y_{n}^{m}(\theta ,\varphi )$$

(2) $$Y_{n}^{m}(\theta ,\varphi )=\sqrt{\frac{(2n+1)(n-m)!}{4\pi (n+m)}}P_{n}^{m}(cos\theta )e^{im\varphi }$$

(3) $$P_{n}^{m}(x)=\frac{{\left(-1\right)}^{m}}{2^{n}n!}\;{\left(1-x^{2}\right)}^{m/2}\frac{d^{n+m}}{{dx}^{n+m}}{\left(x^{2}-1\right)}^{n}$$

(4) $${{(C}_{nj}^{m})}^{T}=u_{B}+\sigma_{B}^{2}y=u_{B}+\sqrt{\lambda }V_{PC}y$$

(5) $$b_{nj}=u_{B}+\chi_{a}\sum_{t=1}^{20}b_{t}+\chi_{b}\sum_{t=21}^{30}b_{t}+\chi_{c}\sum_{t=31}^{73}b_{t}+\cdots$$

where r(θ,φ) is the polar radius; $Y_{n}^{m}(\theta ,\varphi )$ is the SH function; θ and φ are the spherical coordinates zenith angle and azimuth angle; $P_{n}^{m}(x)$ is the associated Legendre function; n and m are the frequency and the order; $B={\left[real(C_{nj}^{m}),imag(C_{nj}^{m})\right]}^{T}$ indicates the whole sample, real(*) and imag(*) extract the real and imaginary parts; $u_{B}$ and $\sigma_{B}^{2}$ are the mean and the variance of **B**; y is the variation coefficients; $V_{PC}$ and λ are eigenvector and eigenvalue, respectively, after principal component analysis (PCA) of $\sigma_{B}^{2}$; $b_{t}=y_{t}\sqrt{\lambda_{t}}v_{PCt}$ is the original particle; and $\chi_{a}$ and $\chi_{c}$ are the scaling factors which are responsible for general form (GF) and local roundness (LR). Note that the PC divisions for different length scales are obtained from the structure of the PC matrix and have been verified by a parametric study of scaling factors at various length scales [40].

### 2.3. DEM Modelling

**Figure 2:**
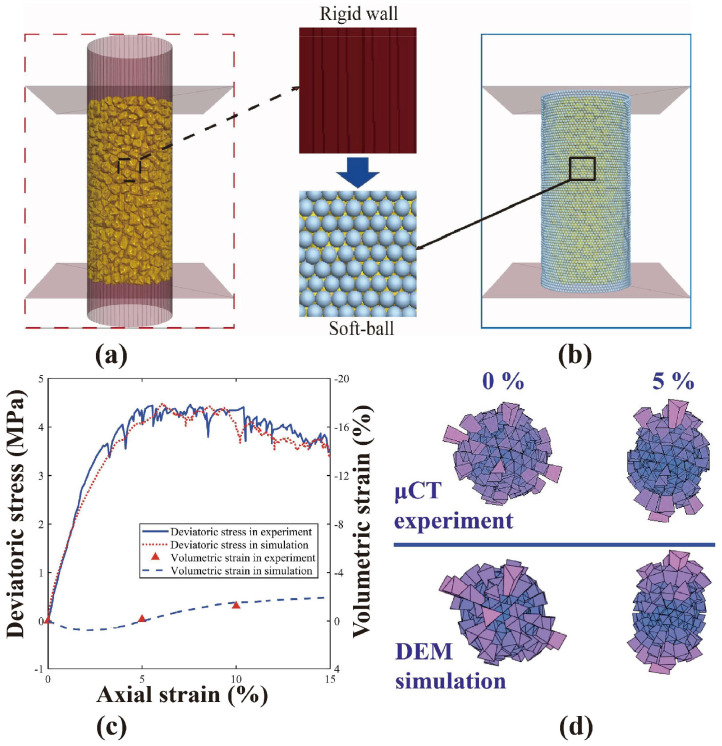
Model calibration between the μCT experiment and one-to-one mapping sample: (**a**) Rigid-wall servo-control system; (**b**) bonded-ball servo-control system; (**c**) deviatoric stress-volumetric strain-axial strain response; and (**d**) 3D rose map of branch vectors.

To simplify and quantify the influence of PM on DEM simulations in reflecting the coupled effect of PSD-PM on the micro-macro mechanical responses of granular soils, one particle was chosen from the physical sample to generate derivative particles with different degrees of morphological gene mutation. Note that morphological gene mutation is based on the PCA of $\sigma_{B}^{2}$; thus, all the experimental particles are involved. By varying $\chi_{a}$ and $\chi_{c}$ in Equation (5) from 0 to 1.5, derivative particles with different degrees of morphological gene mutation can be generated. After incorporating into PFC3D, virtual samples with different particle morphology are reconstructed. A two-step servo-control system was adopted, i.e., the conventional rigid-wall servo-control system (Figure 2a) to load the sample to predefined loading conditions, and the bonded-ball servo-control system to simulate the latex membrane utilised in the experiment (Figure 2b). The corresponding external equivalent force on each membrane ball is updated in every 100 timesteps by Equation (6).

(6) $$F_{e}=\frac{\sigma_{3}}{3}\sum_{i}^{6}n_{i}S_{Ai}$$

where $\sigma_{3}$ is the confining stress, and $S_{Ai}$ and $n_{i}$ are the surface area and the unit normal vector of the *i*th neighbouring triangle. During triaxial tests, the top and bottom platens were loaded with a constant velocity of 0.005 m/s to an ultimate axial strain of 30%.

**Figure 3:**
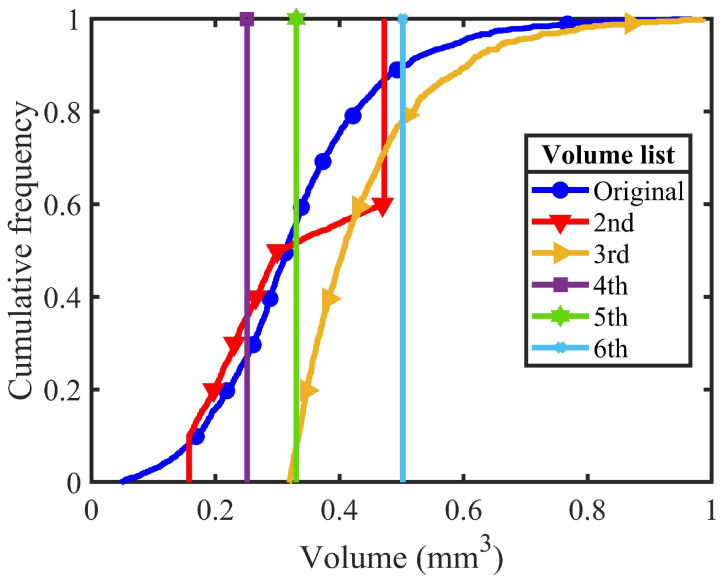
Different types of particle size distribution.

We utilised a one-to-one mapping sample with the same particle morphologies, volumes, and spatial locations as the experimental sample to calibrate the model parameters. Table 1 shows the calibration results, which agreed well with Wu et al. [68] and de Bono and McDowell [73]. The deviatoric stress-volumetric strain-axial strain curve (Figure 2c) and 3D rose map of branch vectors (Figure 2d) were compared between the one-to-one mapping sample and the physical experiment [68,74]. The excellent agreement between simulation and experimental results indicates the feasibility and reasonability of the chosen DEM model parameters.

### 2.4. Virtual Sample Description

In total, 25 DEM samples with different PSDs and morphological gene mutation degrees were generated to conduct virtual triaxial tests. Specifically, 18 DEM samples were generated containing all other combinations of six PSDs and three morphological gene mutation degrees; 6 DEM samples were formed by the chosen realistic particle with six different PSDs; and the one-to-one mapping sample was generated for the model calibration. Figure 3 and Table 2 show the details of the 6 PSDs in this study. Here, the original volume list is extracted from the physical experiment. The original, 2nd, and 5th volume lists share similar particle numbers and mean particle volume V50 but show different uniformity coefficients Cu with a range from 1 to 2.98. In contrast, the 4th, 5th, and 6th volume lists utilise one particle volume for all the particles; thus, all uniformity coefficients equal 1. The corresponding particle numbers vary from 1000 to 2000, while the mean particle volumes vary from 0.25 to 0.5 mm^3^. Besides, the 3rd volume list removes 383 small particles from the original volume list, and then slightly enlarges the remaining particles to guarantee a similar sample volume.

**Figure 4:**
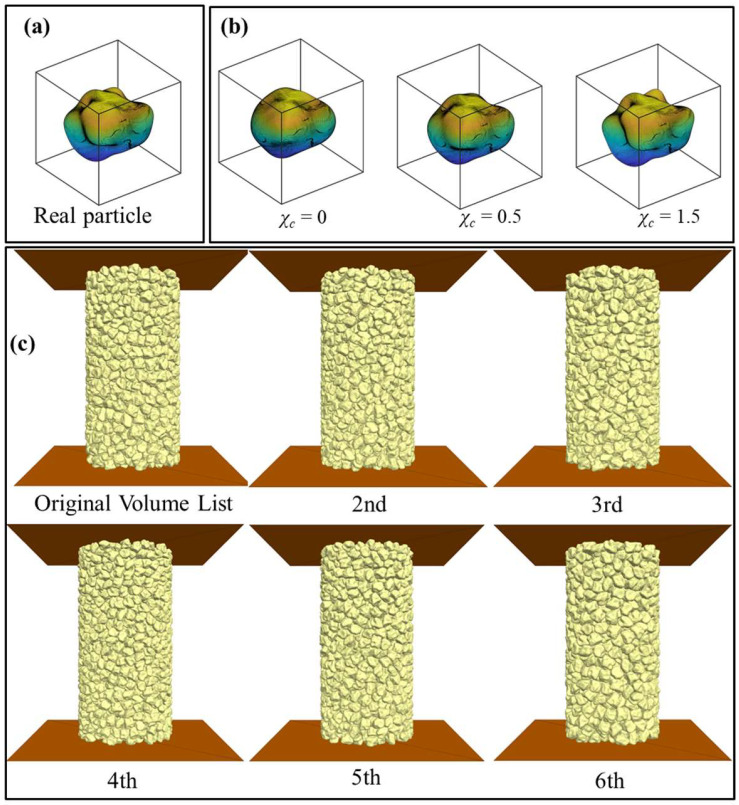
Different degrees of morphological gene mutation and particle size distribution (PSD): (**a**) real particle; (**b**) morphological gene mutation; and (**c**) DEM samples with different PSDs.

Furthermore, 3 degrees of morphological gene mutation at LR-level (GM-LR) were considered to generate derivative particles, i.e., $\chi_{c}$ = 0, 0.5, and 1.5. Figure 4a visualises the chosen real particle, and the corresponding morphological gene-mutated derivative particles are shown in Figure 4b. As observed, the increasing $\chi_{c}$ leads to a rougher particle surface. The resulting DEM virtual samples that consider different PSD and PM are then generated and visualised in Figure 4c. To quantificationally investigate the effects of PM, six traditional morphology descriptors are adopted, i.e., the aspect ratio for GF; roundness for LR; and sphericity and convexity as the overall shape parameters for all length scales [27,75]. The descriptors for the four particles are shown in Table 3. GM-LR witnesses a smaller roundness with increasing $\chi_{c}$ from 0 to 1.5, while not affecting the aspect ratio.

## 3. Results and Discussion

### 3.1. Deviatoric Stress Against Axial Strain

**Figure 5:**
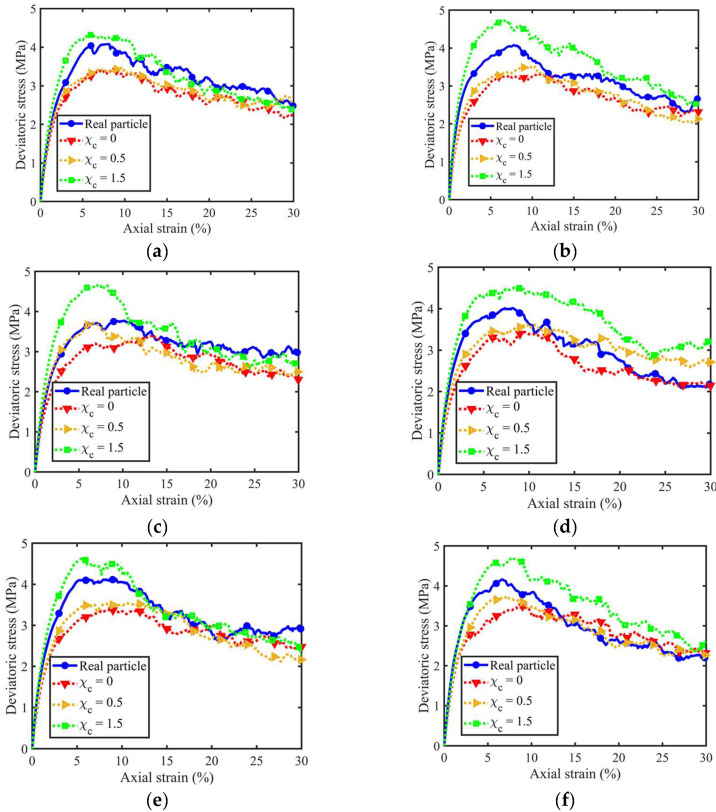
Stress–strain curves for different PSD and morphological variance: (**a**) original volume list; (**b**) 2nd volume list; (**c**) 3rd volume list; (**d**) 4th volume list; (**e**) 5th volume list; and (**f**) 6th volume list.

Figure 5 illustrates the coupled effect of PSD-PM on the macroscopic deviatoric stress *q* vs. axial strain $\varepsilon_{l}$ relationship. Here, *q* and $\varepsilon_{l}$ are defined as Equations (7) and (8):

(7) $$q=\sigma_{1}-\sigma_{3}$$

(8) $$\varepsilon_{l}=\frac{h_{0}-h}{h_{0}}$$

where $\sigma_{1}$ and $\sigma_{3}$ are the axial and confining stresses, and $h_{0}$ and h are the heights at the initial and deformed states. From Figure 5, all curves share a consistent evolution trend, regardless of different PSDs and morphological gene mutation degrees. They witnessed an increase in deviatoric stress to the peak state at an axial strain of around 7% and then showed a mild decrease to the critical state. In terms of the morphological gene mutation, the two samples with $\chi_{c}$ = 0 and 1.5 form the lower and upper bounds of the initial stiffness, peak-state, and critical state deviatoric stresses, and a larger $\chi_{c}$ leads to a more pronounced value. This is because particles with $\chi_{c}$ = 1.5 have more sharp corners and edges, impeding the particles within the given sample from rotating and translating during triaxial shear. Furthermore, the difference between the lower and upper bounds at peak-state is more significant than that at the critical state, indicating that the PM at LR-level shows fewer effects on the critical-state stress–strain behaviours.

**Figure 6:**
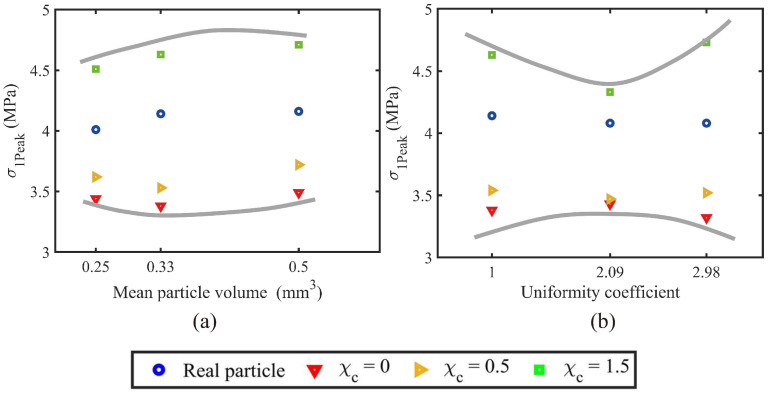
Coupled effects of particle size distribution and morphological variance on peak deviatoric stress: (**a**) mean particle volume; and (**b**) uniformity coefficient.

Furthermore, the particle size and PSD uniformity show limited effects on the evolution trend of deviatoric stress-axial strain curves. To better visualise the contribution of particle size and PSD uniformity to the PM effect, all the peak-state deviatoric stresses for different PSD and morphological gene mutations were extracted, as shown in Figure 6. Figure 6a considers the 4th, 5th, and 6th volume lists, and their uniformity coefficients equal 1. In contrast, Figure 6b compares the original, 2nd, and 5th volume lists, and these three samples share a similar mean particle volume but different uniformity coefficients. As can be observed, the increased mean particle volume will slightly increase the peak-state deviatoric stress, and this phenomenon is more evident in rougher particles with $\chi_{c}$ = 1.5. Moreover, the contribution of mean particle volume to the PM effect slightly increases and remains constant, increasing the mean particle volume from 0.25 to 0.50 mm^3^. Compared to the mean particle volume, the uniformity of PSD shows a more notable influence in the PM effect. The difference between peak-state deviatoric stresses of the roughest and smoothest samples is sharply narrowed with the uniformity coefficient varying from 1 to 2.09 and then enlarged with the continuously increased uniformity coefficient. Thus, the uniformity of PSD shows more contribution than the mean particle volume to the PM effect of granular soils.

### 3.2. Volumetric Strain Evolution

**Figure 7:**
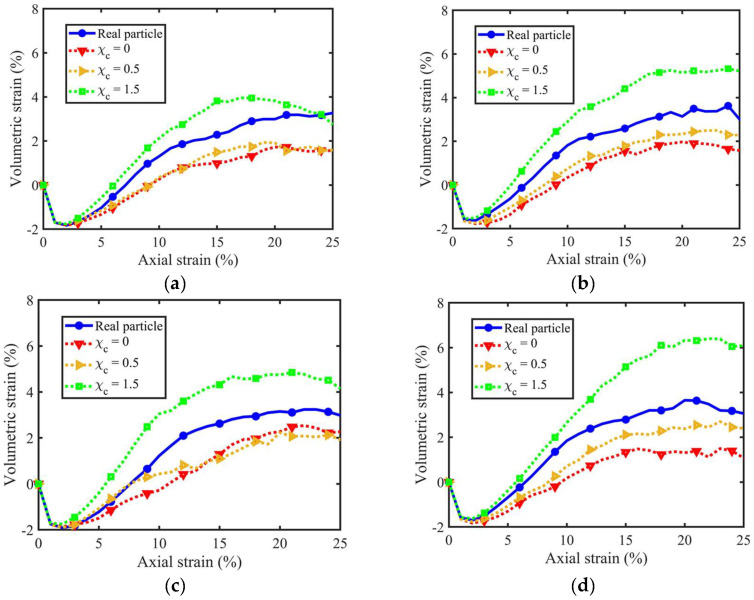

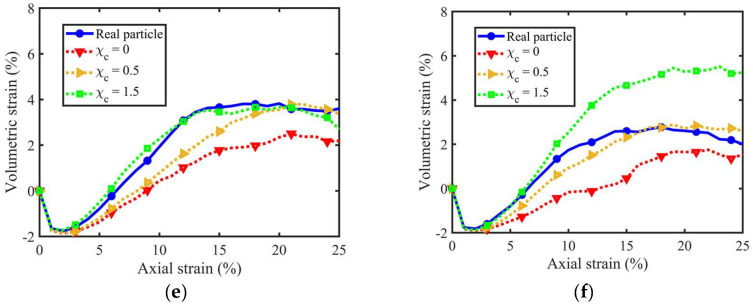
Volumetric strain curves for different PSD and morphological variance: (**a**) original volume list; (**b**) 2nd volume list; (**c**) 3rd volume list; (**d**) 4th volume list; (**e**) 5th volume list; and (**f**) 6th volume list.

Figure 7 shows the volumetric strain variations vs. axial strain considering different PSDs and morphological gene mutation degrees. The volumetric strain $\varepsilon_{V}$ is defined as follows:

(9) $$\varepsilon_{V}=\frac{∆V}{V_{0}}={2\varepsilon }_{R}+\varepsilon_{l}+\varepsilon_{R}^{2}+{2\varepsilon }_{R}\varepsilon_{l}+\varepsilon_{R}^{2}\varepsilon_{l}$$

where $\varepsilon_{R}$ and $\varepsilon_{l}$ are the radial and the axial strains expressed by $\varepsilon_{R}=∆R/R_{0}$ and $\varepsilon_{l}=∆h/h_{0}$; ∆V, ∆R, and ∆h are the increments of the sample volume, average membrane radius, and sample height; and $V_{0}$, $R_{0},\;$ and $h_{0}$ denote the corresponding values at the initial state. Generally, all curves experienced a similar variation trend: an initial contraction and then a significant dilation, which gradually diminishes to a constant volume at the critical state. The larger $\chi_{c}$ led to a slightly smaller initial contraction, higher maximum dilation angle, and more prominent ultimate volumetric strain.

**Figure 8:**
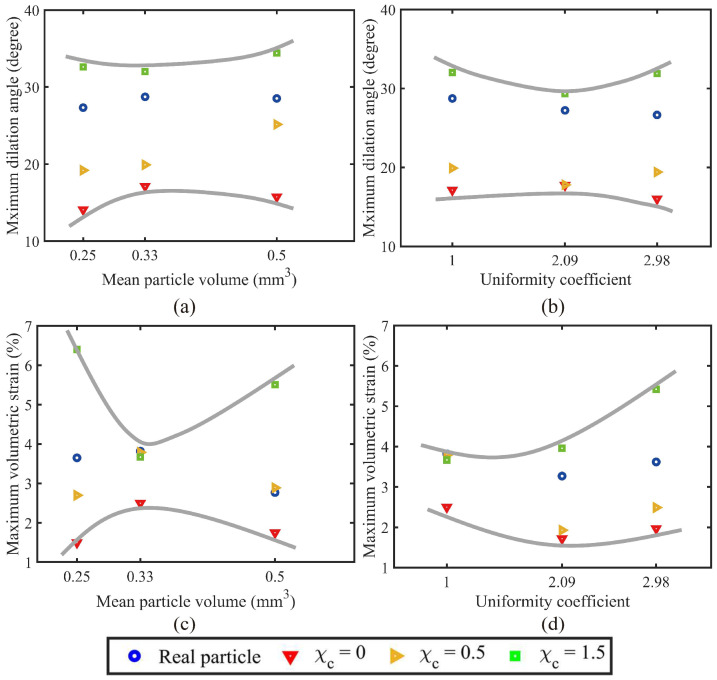
Coupled effects of particle size distribution and morphological variance on (**a**,**b**) maximum dilation angle and (**c**,**d**) maximum volumetric strain.

Furthermore, samples with different PSDs show a similar evolution trend among different morphological gene mutation degrees, implying that PSD uniformity has limited influence on the evolution of volumetric strain. Figure 8 extracts the maximum dilation angle and the maximum volumetric strain of each sample to visualise the contributions of the particle size and PSD uniformity to the PM effects of granular soils. Here, Figure 8a,c considers the 4th, 5th, and 6th volume lists with the same uniformity coefficient and different mean particle volumes. Figure 8b compares the original, 2nd, and 5th volume lists with similar mean particle volume but different uniformity coefficients. As observed, the mean particle volume and the uniformity coefficient show limited influence in the evolution of maximum dilation angle, with minor variations among different morphological gene mutation degrees. In contrast, the maximum volumetric strain witnesses a notable difference among different degrees of morphological gene mutation. The mean particle volume sees first a decreased and then an increased contribution to the PM effects, while the PSD uniformity witnesses a continuously increased contribution.

### 3.3. Coordination Numbers

**Figure 9:**
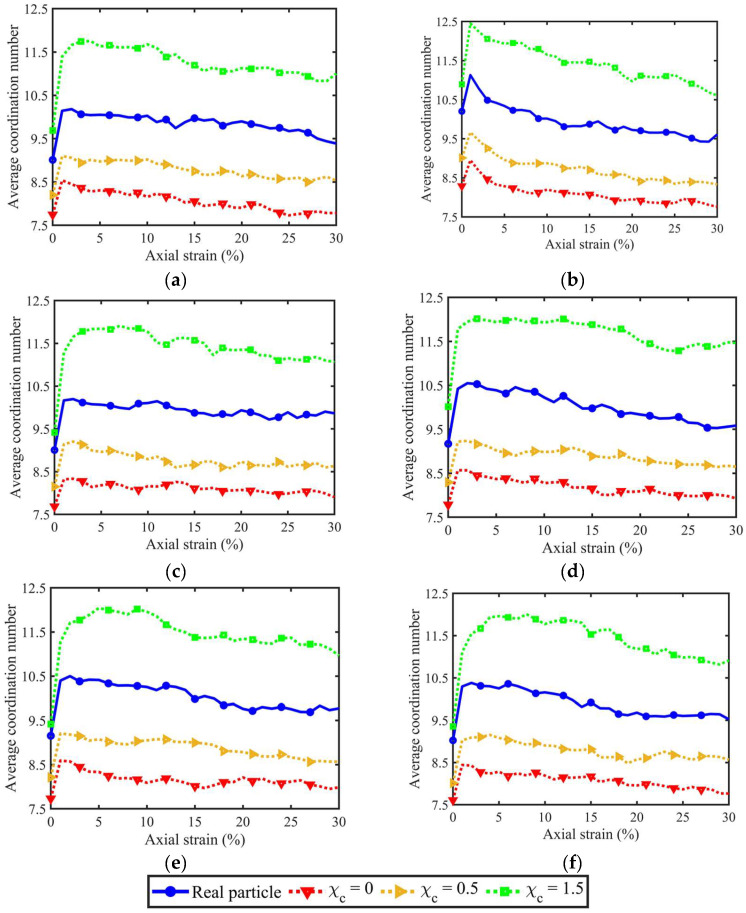
Contact-based coordination numbers for different PSD and morphological variance: (**a**) original volume list; (**b**) 2nd volume list; (**c**) 3rd volume list; (**d**) 4th volume list; (**e**) 5th volume list; and (**f**) 6th volume list.

**Figure 10:**
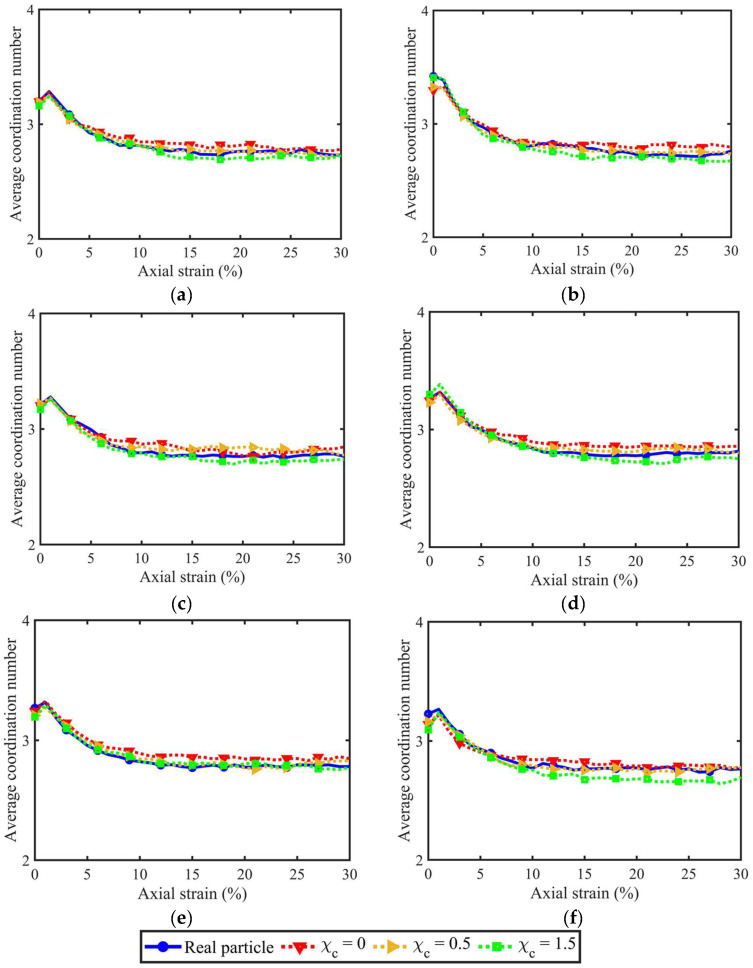
Particle-based coordination numbers for different PSD and morphological variance: (**a**) original volume list; (**b**) 2nd volume list; (**c**) 3rd volume list; (**d**) 4th volume list; (**e**) 5th volume list; and (**f**) 6th volume list.

The coordination number (CN) can be categorised into two types, i.e., contact-based CN and particle-based CN. The former CN is the number of contacts belonging to the specific particle, while the latter CN is the number of particles contacting the particle. The rough surfaces of particles within the given sample usually lead to multiple contacts, instead of one, between two particles in touch. Hence, the two types of CN usually have different values. Figure 9 shows the evolution of contact-based CN considering different PSDs and morphological gene mutation degrees. GM-LR has significant effects on the development of contact-based CN. This is because rough PM at LR-level will increase the sharp corners or edges, which can augment the number of contacts per particle. In contrast, the particle-based CN in Figure 10 shows a consistent variation trend among different morphological gene mutation degrees, indicating that PM at LR-level has limited effects on the evolution of particle-based CN.

Figure 10d–f shares the same PSD uniformity but different mean particle volumes. As observed, all three volume lists witness a similar evolution trend of contact-based CN, indicating that the mean particle volume has little effect on the contact-based CN. This is because, for any particle within the sample, this particle and its neighbouring particles are enlarged to the same degree, and the contacts belonging to this particle will not significantly change. Furthermore, Figure 10a,b,e has similar mean particle volumes but different PSD uniformity coefficients from 1 to 2.98. As can be observed, the increasing uniformity coefficient augments the maximum contact-based CN, showing that the PSD uniformity contributes a lot to the evolution of contact-based CN. This is because the PSD uniformity changes the percentages of larger and smaller particles, and the increasing size creates more contacts for each large particle.

### 3.4. Fabric Evolution

**Figure 11:**
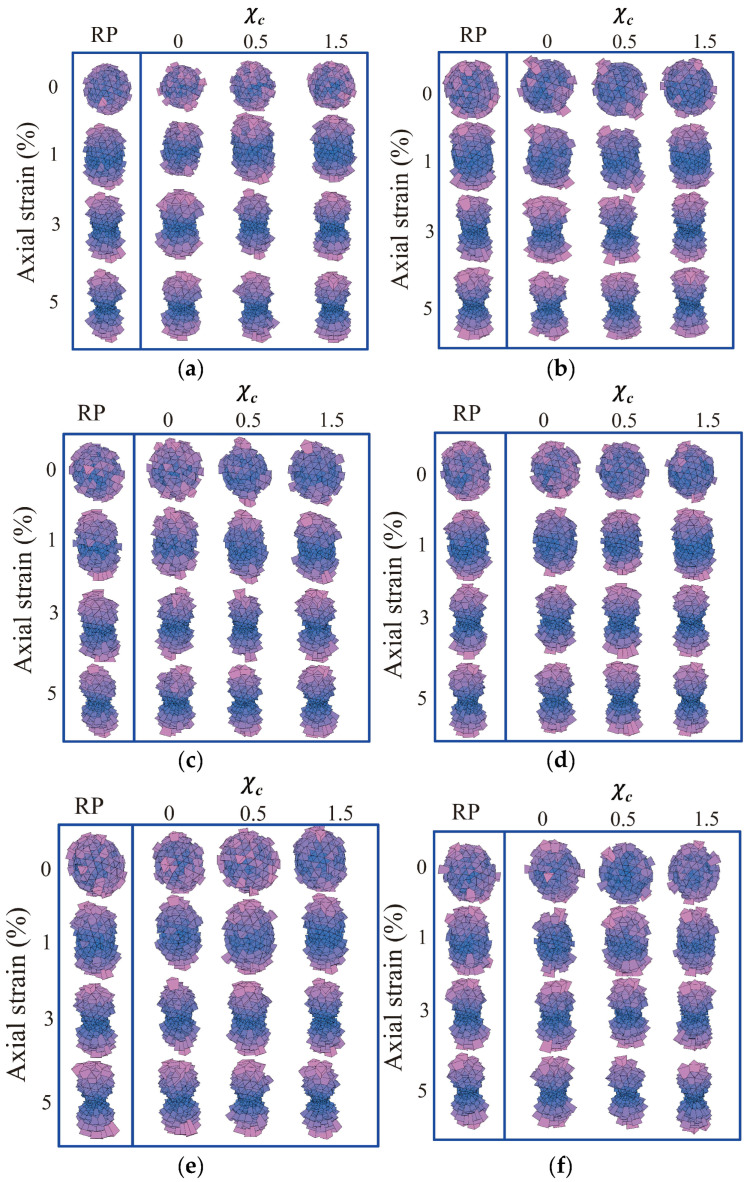
Fabric evolution for different morphological variance degrees and particle size distributions: (**a**) original volume list; (**b**) 2nd volume list; (**c**) 3rd volume list; (**d**) 4th volume list; (**e**) 5th volume list; and (**f**) 6th volume list.

Figure 11 shows the fabric evolution of granular soils considering different PSDs and PMs. Here, the 3D rose map for each case counts all the normal vectors of the contact forces between particles within the given sample. Generally, regardless of the PSD or morphological gene mutation degree, all the samples show a dispersive distribution of contact normal directions at the axial strain of 0%, reflecting that all the initial samples are in a roughly isotropic state. With the ongoing triaxial shear, the contact in normal directions gradually converges with the direction of the major principal stress. Among different degrees of morphological gene mutation, smoother particles with a smaller $\chi_{c}$ witness a later convergence of contact normal directions to the direction of major principal stress than rougher particles with a larger $\chi_{c}$. This is because smoother particles are more likely to translate and rotate under external loading than rougher particles, and hence it takes more time for smoother particles to settle to a steady state. Furthermore, with the same PSD uniformity coefficient but different mean particle volumes, Figure 11d–f illustrates a similar evolution of contact normal directions among different volume lists. This phenomenon implies that the particle size has limited effects on the fabric evolution of granular soils with unchanged PSD uniformity and PM. Moreover, Figure 11a,b,e shares a similar mean particle volume but a varying PSD uniformity coefficient. As observed, the contact normal directions in the sample with a smaller PSD uniformity coefficient witness a later convergence to the direction of major principal stress. For instance, the sample with $\chi_{c}$ = 0 and Cu = 1 (in Figure 11e) witnesses an early convergence to the vertical direction at an axial strain of 1% compared to the sample with the same $\chi_{c}$ and a larger Cu (see Figure 11a,b).

### 3.5. Shear Band Evolution

**Figure 12:**
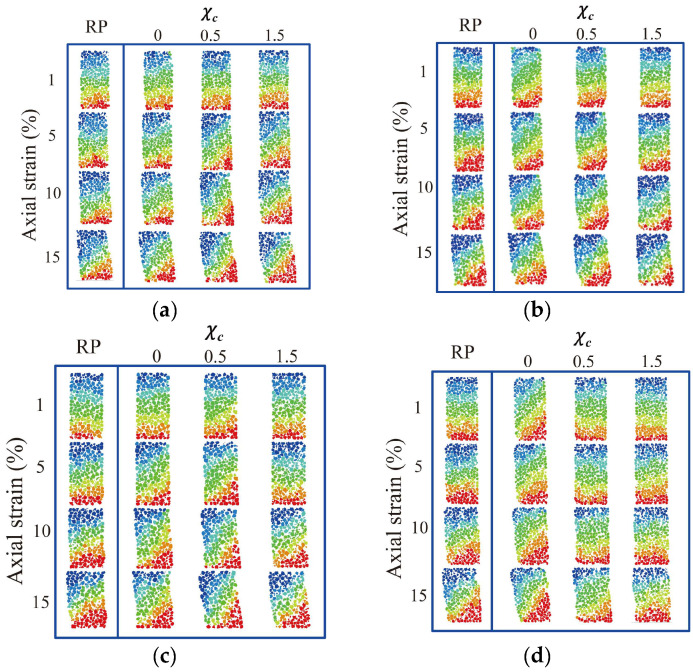

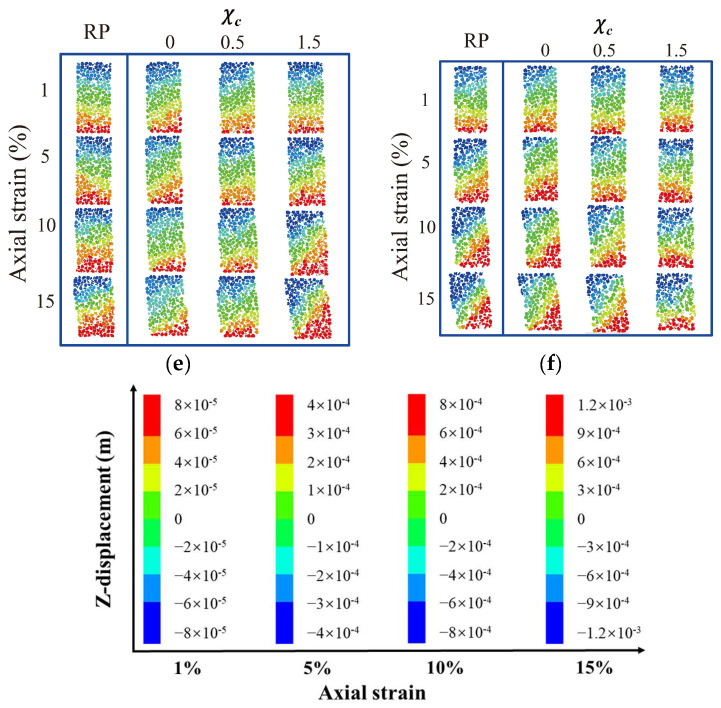
Shear band evolution for different morphological variance degrees and particle size distributions: (**a**) original volume list; (**b**) 2nd volume list; (**c**) 3rd volume list; (**d**) 4th volume list; (**e**) 5th volume list; and (**f**) 6th volume list.

Figure 12 shows the shear band evolution of granular soils with a consideration of different PSDs and PMs. Here, the vertical displacement of each particle was extracted and utilised to visualise the shear band formation. Furthermore, snapshots of four representative stages, i.e., 1%, 5%, 10%, and 15%, were chosen to describe the evolution of the shear band. Note that all the samples share the same displacement intervals for specific axial strains. As can be observed from Figure 12, all the samples illustrate evident inclined shear bands at the axial strain of 15%. However, the shear band formation processes, i.e., the shape, thickness, and orientation of the shear band, differ among different morphological gene-mutated samples. Specifically, at the axial strain of 1%, the samples with $\chi_{c}$ = 0 exhibit the earliest formation of the shear band compared to the samples with a larger $\chi_{c}$, indicating that the smoother particles lead to earlier formation of the shear band compared to the rougher particles with a larger $\chi_{c}$. This is because particles with smoother particles are easier to translate and rotate, leading to an earlier formation of an inclined shear band. At an axial strain of 15%, the samples with $\chi_{c}$ = 0 exhibit a more inclined final sample shape and shear band direction than the samples with a larger $\chi_{c}$. From Figure 12d–f, the samples with the same PSD uniformity coefficient but different mean particle volumes witness a very similar formation trend of the shear band among different volume lists. With a similar mean particle volume but a varying PSD uniformity coefficient, the sample with Cu = 1 and $\chi_{c}$ = 0.5 (Figure 12e) shows an early formation of the shear band compared to the samples with the same $\chi_{c}$ and a larger Cu. This phenomenon implies that the PSD uniformity can accelerate the shear band formation.

## 4. Conclusions

This study utilised a novel tomography-based DEM technique, including grain-scale property extraction by the X-ray μCT, morphological variation by SH-PCA, DEM parameter calibration by the one-to-one mapping technique, and massive derivative DEM simulations. Six PSDs and four PMs were adopted, and in total 25 DEM samples were generated. The effects of PSD and PM on the micro-macro mechanical behaviours of granular soils were carefully investigated, and the coupled effects of both were highlighted. The main contributions and findings are summarised as follows.

(1) It was found that particle morphology plays a significant role in deviatoric stress vs. axial strain, volumetric strain vs. axial strain relationships, coordination numbers, fabric evolution, and shear band evolution. Specifically, the rougher particle surface witnesses the more pronounced values of the initial stiffness, peak deviatoric stress, maximum dilation angle, ultimate volumetric strain at critical state, and contact-based CN. Furthermore, the rougher particle surface leads to early convergence of contact normal directions to the direction of major principal stress, and later formation of the shear band.
(2) The uniformity of particle size distribution can enhance the particle morphology effect of granular soils in dictating the peak deviatoric stress, maximum volumetric strain, contact-based coordination number, fabric evolution, and shear band formation. Limited influences can be found in the maximum dilation angle and particle-based coordination number. Furthermore, with the same particle size distribution and particle morphology uniformity, the mean particle volume shows very small effects on the macro-micro mechanical behaviours of granular soils.

These results highlight the role of particle morphology and particle size distribution on the micro-macro mechanical behaviours of granular soils. The following limitations are acknowledged: (1) only morphological variance at LR-level is considered to simplify the issue; (2) the uniformity coefficients of the current six PSDs are still not in a large range. Future studies will consider morphological variance at more length scales and a more extensive range of PSD uniformities.

## Figures and Tables

**Table 1:** DEM model parameters.

Groups	Items	Value
Sample parameters	Size: height × diameter (mm)	16 × 8
	Number of particles	1520
	Density (kg/m^3^)	2650
	Poisson’s ratio	0.25
	Damping coefficient	0.7
	Radius ratio of the smallest to largest sphere	0.3
	Sphere-to-sphere intersection angle (degree)	150
	Clump friction	0.3
	Shear modulus (GPa)	28
	Contact model	Hertz-Mindlin
Membrane parameters	Number of balls	7600
	Density (kg/m^3^)	1000
	Tensile strength (N)	1 × 10^200^
	Tangential strength (N)	1 × 10^200^
	Normal stiffness (N/m)	1 × 10^6^
	Shear stiffness (N/m)	1 × 10^6^
	Ball radius (mm)	0.128

**Table 2:** Details of different particle size distributions.

PSD	Number of Particles	Mean Particle Volume (V50) (mm^3^)	Uniformity Coefficient (*C_u_* = *V*_60_/*V*_10_)
Original volume list	1520	0.31	2.09
2nd volume list	1500	0.33	2.98
3rd volume list	1137	0.41	1.29
4th volume list	2000	0.25	1
5th volume list	1520	0.33	1
6th volume list	1000	0.50	1

**Table 3:** Morphology descriptors.

$\chi_{c}$	Aspect Ratio	Roundness	Sphericity	Convexity
0	0.912	0.731	0.854	0.952
0.5	0.912	0.672	0.844	0.937
1	0.912	0.582	0.815	0.899
1.5	0.912	0.554	0.775	0.848

## Data Availability

The original contributions presented in this study are included in the article. Further inquiries can be directed to the corresponding author.
